# Diffuse Pancreatic Carcinoma with Hepatic Metastases

**DOI:** 10.1155/2020/8815745

**Published:** 2020-10-30

**Authors:** Hoang Quan Nguyen, Ngoc Trinh Thi Pham, Van Trung Hoang, Hoang Anh Thi Van, Chinh Huynh, Duc Thanh Hoang

**Affiliations:** ^1^Department of Radiology, Da Nang Oncology Hospital, Da Nang, Vietnam; ^2^School of Medicine and Pharmacy, The University of Danang, Da Nang, Vietnam; ^3^Department of Radiology, Thien Hanh Hospital, Buon Ma Thuot, Vietnam; ^4^Department of Radiology, Tu Du Hospital, Ho Chi Minh, Vietnam; ^5^Division of Endocrinology, Department of Medicine, Walter Reed National Military Medical Center, Bethesda, USA

## Abstract

Pancreatic cancer is one of the seven leading causes of cancer death worldwide. Diffuse pancreatic carcinoma is very rare and underreported in the literature. Many advances have been made in the diagnosis and management of pancreatic cancer. However, most pancreatic cancer cases are detected at the terminal or metastatic stages. Therefore, timely diagnosis and therapeutic management are desirable goals for this disease. Although the proliferation of pancreatic cancer has been reduced by intervention, more work is needed to treat and prevent the disease. The purpose of this article is to present a case of a 54-year-old male with pancreatic cancer and to review the epidemiology, diagnosis, management, and prevention of pancreatic tumors in general as well as pancreatic carcinoma in particular.

## 1. Introduction

Pancreatic cancer can derive from epithelial and nonepithelial tumors such as pancreatic metastases, connective cancer, and lymphoma. Epithelial tumors include nonendocrine and endocrine tumors such as pancreatic ductal adenocarcinoma, serous cystadenocarcinoma, and mucinous cystadenocarcinoma [1]. Among all cancers, pancreatic cancer is one of the worst; despite improvements in both diagnostic and surgical techniques, survival has not improved substantially for more than 50 years. The 5-year survival rate still remains at 9% only. Pancreatic carcinoma can occur at any age but is more common in the elderly. Pancreatic cancer is the 11th most common cancer in the world, accounting for 2.5% of all cancers and causing 4.5% of all cancer deaths [2]. Due to the increasing incidences, late diagnosis, and limited treatment options, its mortality rate is estimated to increase gradually over the next decade [3]. The tumor's response to chemotherapy and radiation is poor. Surgery is the only treatment that has the potential to prolong survival but is usually performed late at the time of discovery [4]. Diffuse pancreatic carcinoma is usually rare compared to focal pancreatic cancer. In a major study of general pathology, the location of pancreatic carcinoma at the head of the pancreas was 60%, the pancreatic body 13%, pancreatic tail 5%, and the remaining 22% diffuse pancreatic carcinoma [2, 5, 6]. Biochemical testing and diagnostic imaging are the main diagnostic methods [4]. Differential diagnosis includes diffuse pancreatitis, autoimmune pancreatitis, multiple pancreatic cancer, and diffuse pancreatic metastases. Distinguishing between pancreatic tumors and pancreatitis is crucial because the clinical course, treatment, and prognosis of these two diseases are quite different [5, 7]. Metastatic pancreatic carcinoma mainly affects the liver, peritoneum, lung, and bone, although bone metastases usually occur late [5, 8, 9]. In this case report, we describe a rare case of a 54-year-old male with diffuse pancreatic carcinoma with hepatic metastases diagnosed by ultrasound, computed tomography (CT), and magnetic resonance imaging (MRI).

## 2. Case Presentation

A 54-year-old man was hospitalized with diffuse abdominal pain and abdominal distention. The pain occurred 4 days prior to admission and was gradually increasing. At first, the pain was localized in the epigastric region, then radiating throughout the abdomen. The patient reported nausea but no vomiting nor jaundice. He was normotensive with a blood pressure of 120/60 mmHg, pulse rate 75 bpm, respiratory rate 21 per minute, and temperature 37.7°C. He noticed recent weight loss of 4 kg over 3 months. He reported rare alcohol consumption, no smoking, and noncontributing family history. Examination showed no symptoms of biliary obstruction but diffuse abdominal tenderness on palpation. Serum amylase, lipase, and IgG4 were within normal ranges, and CA19-9 and CA242 were 69.6 U/mL (normal value ≤ 37 U/mL) and 82.1  U/mL (normal value ≤ 20 U/mL), respectively. Bilirubin, alpha-fetoprotein, and HBsAg were normal.

**Figure 1:**
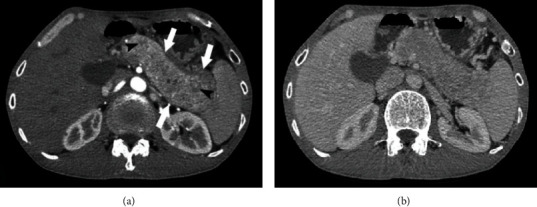
Contrast-enhanced CT images in the arterial (a) phase and delayed (b) phase. CT shows a sausage-shape enlargement of the pancreas (arrows), with relatively homogenously remarkable enhancement in the arterial phase and washout in the delayed phase with the capsule-like rim at the surface (arrowheads). The pancreatic duct did not dilate.

**Figure 2:**
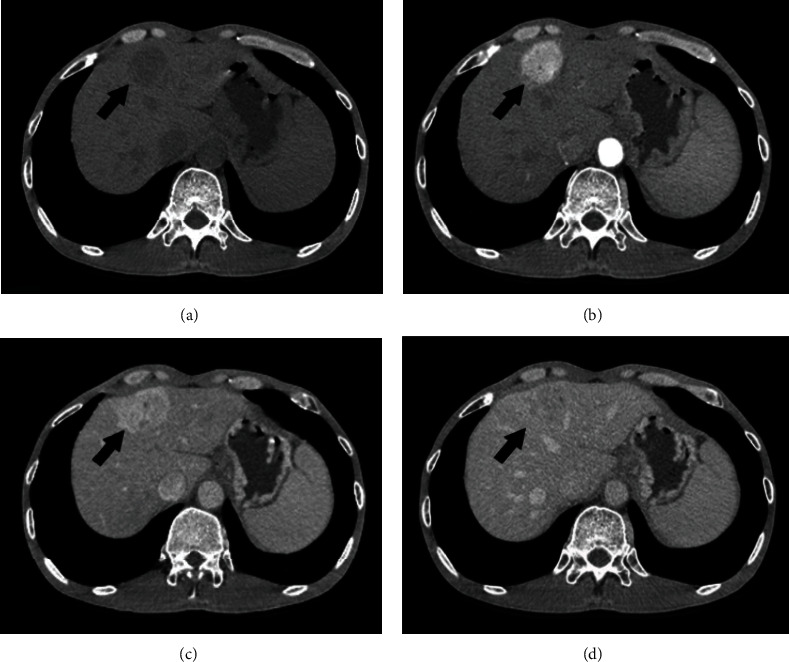
CT images before (a) and in the late arterial (b), portal venous (c), and delayed (d) phases after contrast administration. The metastases hyper enhance dramatically in the arterial phase and subsequently fade toward mild hypoattenuation relative to the liver in the delayed phase (arrows). Some portions of the lesions do not enhance, suggesting necrosis. Many similar lesions are scattered throughout the liver parenchyma (not shown).

**Figure 3:**
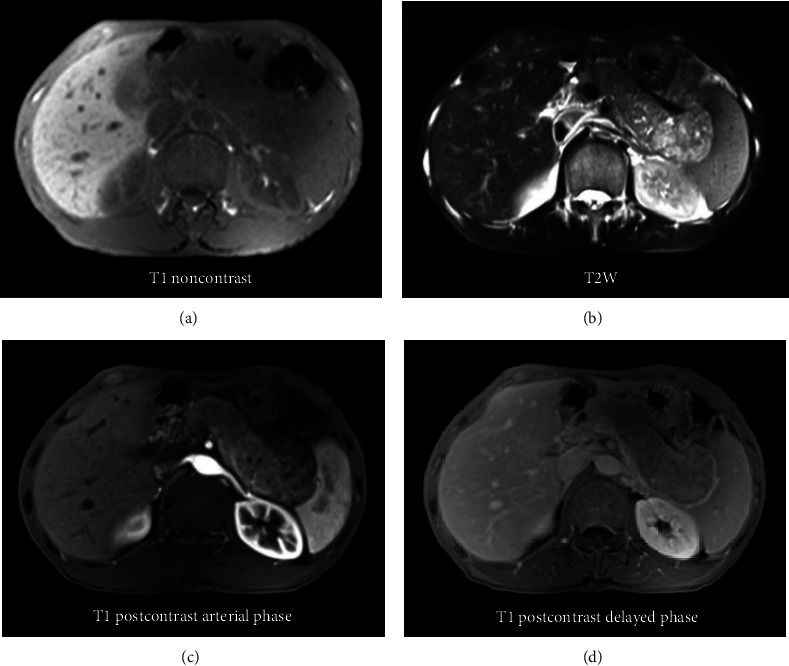
MRI on T1-weighted image (a), T2-weighted image (b), and arterial phase (c) and delayed phase (d) in T1-weighted image with fat-sat after contrast administration. MRI shows a diffusely enlarged pancreas with low intensity on T1-weighted imaging and heterogeneous high intensity on T2-weighted imaging, particularly in the body and tail region of the pancreas. The pancreas also shows remarkable enhancement in the arterial phase and washout in the delayed phase.

Abdominal ultrasound revealed a diffusely enlarged and heterogeneous hypoechoic pancreas. Slightly infiltrated fat layer was found around the pancreas without associated fluid. Simultaneously, the liver also showed multiple solid hypoechoic or hyperechoic masses. CT scan confirmed enlargement of the pancreas with relatively homogenous enhancement remarkably in the arterial phase and washout in the delayed phase (Figure 1). Multiple liver lesions showed early arterial enhancement with washout in the delayed phase suggesting hypervascular metastases (Figure 2). The pancreatic duct and biliary tract did not dilate; there was no presence of pathological lymph nodes. The patient subsequently underwent MRI that showed characteristics similar to ultrasound and CT with a diffusely enlarged pancreas with low signal intensity on T1-weighted, high signal intensity heterogeneous on T2-weighted, restricted diffusion on diffusion-weighted imaging, and relatively homogenous enhancement on postcontrast T1-weighted images (Figure 3).

According to the clinical presentation, laboratory results, and radiological findings, we had a provisional diagnosis of diffuse pancreatic carcinoma with liver metastasis. We performed pancreatic FNA under ultrasound guidance; cytology revealed suspected carcinoma. The patient then underwent ultrasound-guided biopsy of liver lesions; histopathological findings confirmed metastases from carcinoma. He received conservative treatment, and after 2 weeks, the symptoms were much improved. We offered chemoradiotherapy and palliative care, but he refused and requested to be discharged 1 week later. We contacted the patient after 2 months, but family members said his conditions were getting worse.

## 3. Discussion

Pancreatic cancer poses a significant diagnostic challenge, and the majority of cases present late with either locally advanced or metastatic disease. Serum marker CA19-9 has been evaluated with a sensitivity of 70-90% and specificity of 68-91%. Its main value is to assess the response to the treatment and monitor follow-up recurrence of the disease [4, 5, 9]. A novel array set of biomarkers for diagnostic, predictive, and prognostic potential for pancreatic cancer is currently being used and studied with the hope of finding effective management methods for this challenging disease (Table 1) [10]. Further studies of novel biomarkers for pancreatic cancer subtyping, diagnosis, and treatment response prediction are underway [11].

In patients presenting with pancreatic malignancy, abdominal ultrasound is often used as an initial diagnostic modality [12, 13]. CT scan is used in evaluating the stage of pancreatic cancer with a sensitivity of 89-97% and specificity of 95%. CT findings indicate the presence of the tumor, involvement of vessels (the celiac axis and hepatic or superior mesenteric artery) and organs, enlarged lymph nodes, ascites, and distant metastases. In our case, the peripheral capsule-like structure was a characteristic CT finding of diffuse pancreatic cancer that was rarely seen in focal pancreatic cancer and other pancreatic lesions [5, 8, 12]. MRI is another appropriate option for patients who have contraindications to CT or where CT images are unclear [5, 13]. High resolution of MRI can better detect pancreatic lesions, small liver metastases, lymph nodes, and peritoneal metastases. The recognization of the liver and peritoneal metastases is critical for accurately staging pancreatic carcinoma because the cancer is unresectable if metastases are present [4, 14]. Multislice CT can also detect small liver lesions; however, if the lesions are less than 10 mm, it is often difficult to characterize. MRI may be more clearly delineate lesions like hemangiomas, cysts, or metastases [3]. According to Danet et al. [15], liver metastases have usually signals from isointensity to slightly high intensity on T2-weighted images, with a slightly lower signal on T1-weighted images. Positron emission tomography (PET) is becoming more widely available and may help distinguish chronic pancreatitis from pancreatic cancer. It may help detect regional lymph nodes or distant metastases from pancreatic cancer [4, 8]. Endoscopic ultrasound (EUS) can be used to detect small pancreatic masses that could be missed by CT scan and is commonly used to perform biopsy [6, 9].

The malignancy of the pancreas is classified into resectable, borderline resectable, and advanced disease (either locally advanced or metastatic) [16]. Multidisciplinary treatment of pancreatic carcinoma needs to combine surgery, radiotherapy, chemotherapy, and supportive care. Some current methods include personalized medicine, immunotherapy, innovative targets, therapeutic vaccines, stemness inhibitors, adoptive T-cell transfer, and stem-cell therapy [3, 5, 9]. The stratification of pancreatic cancer patients according to the tumor transcriptome is a major approach to predict treatment response. Other strategies concentrate on genomic or chromosome alterations and the determination of targeted therapies [11, 17].

Comprehensive analyses from genomes of primary and metastatic pancreatic tumors indicate that there are two progressive processes in pancreatic cancer: (1) the early cancer-initiating phase in which most mutations would establish a preneoplastic tumor and (2) the rapid cancer-transforming phase that generates invasive clones and colonization of distant sites. Moreover, the gradual increasing mutational burden of CDKN2A, KRAS, SMAD4, and TP53 genes promotes the early accelerated progression and metastasis of pancreatic cancer. When the biological and molecular landscape of pancreatic carcinoma is unraveling, there would be a shift of clinical practice to utilize molecular taxonomy, where pancreatic cancer would be optimally treated depending on its molecular subtype. Besides, the characterization of cell signaling pathways and the diverse tumor cell types would be useful in the approach to novel therapy [11, 18].

Recent combination therapies are based on mechanisms of resistance to immune checkpoint inhibitors, which improve condition in patients with unresectable pancreatic cancer. According to Porcelli et al. [19], cancer-associated fibroblasts and mast cells reduced the effectiveness of gemcitabine/nab-paclitaxel on inhibition of tumor cells' regrowth. Knowledge of the molecular characteristics of an individual can allow treatment modifications and monitoring of the response to gemcitabine/nab-paclitaxel [19].

Because the pancreatic tumor microenvironment has an important role in the disease progression; therefore, it is a novel area related to targeted therapy that is under active research. More recent therapies including SPARC-targeting are discussed. Studies suggested that SPARC in the tumor stroma may sequester albumin-bound paclitaxel, enhancing the distribution of paclitaxel into the tumor microenvironment. Additionally, some preclinical studies showed an antitumor activity novel agent against PDAC cells. Furthermore, Wnt inhibitors and other novel immunomodulators have been reported to stimulate lymphocytes and innate immunity antitumor response through *γδ*-type T-cell receptors and impact the median time to the first metastasis-related event and overall survival, in which restoring dendritic cells also mediates immunity. Remarkably, innate immunity also seems to be related to zoledronic acid efficacy via tumor-associated macrophages [11, 20].

Challenges in targeted cancer therapy comprising varied resistance mechanisms emerged. They can be caused by the cancer mutational landscape, microenvironment, and inflammation perspectives. Drug resistance could preexist or be acquired during the course of treatment. Alterations in the concentration of chemokines, cytokines, and growth factors have presented as a mechanism of conferring resistance to chemotherapeutic therapies. Anti-inflammatory agents, beyond their ability to decrease or prevent chemotoxicity, can also act synergistically when combined with chemotherapy agents. They may cause sensitization of cancer cells to treatment with conventional anticancer agents. Several studies suggest that anti-inflammatory agents will be most effective in combination with cytotoxic, antiangiogenic, and cytostatic agents. Furthermore, clinical managers would need to identify carefully for each drug the appropriate molecular target, disease stage, cancer type, and treatment duration [18–21].

## 4. Conclusion

Diffuse pancreatic carcinoma is rare and difficult to manage because there are not many samples to study. It is often reported sporadically as our case, with the weakness of no thorough treatment for our patient. This is a challenge that needs more precise approaches and well-designed clinical trials. Researchers need to further investigate other modalities to assess survival and tumor regression. It is necessary to increase the ability to diagnose and detect pancreatic cancer early by various laboratory methods such as serological markers, biomarkers, and cytological markers and radiological modalities such as ultrasound, CT, and MRI. A clinical stratification and screening system combined with prevention techniques and new technologies are needed in both diagnosis and treatment.

## Figures and Tables

**Table 1:** Pancreatic cancer biomarkers.

Diagnostic markers	Carbohydrate antigens, microRNAs, macrophage inhibitory cytokine 1, PAM4 antibodies, glypican, KRAS mutation, osteopontin, epigenetic markers
Predictive biomarkers	Gemcitabine markers, FOLFIRINOX markers, nab-paclitaxel markers, stromal markers, BRCA mutated tumors, microsatellite instability, PD-1/PD-L1
Prognostic markers	CA19-9, SMAD4, angiogenesis markers, inflammatory markers, immune markers, microRNAs, SPARC

## Data Availability

No data were used for this case report.
